# Pars interarticularis repair using pedicle screws and laminar hooks fixation technique in patients with symptomatic lumbar spondylolysis

**DOI:** 10.1051/sicotj/2022013

**Published:** 2022-04-06

**Authors:** Mohammed Zayan, Mohammed Ali Hussien, Hany El Zahlawy

**Affiliations:** Department of Orthopedic surgery, Faculty of Medicine, Ain Shams University 38 Abbassia Square, Next to Al-Nour Mosque Cairo 11865 Egypt

**Keywords:** Pars repair, Pedicle screws, Laminar hooks, Screw-rod-hook fixation, Spondylolysis

## Abstract

*Study Design*: Prospective case series. *Purpose*: To assess the outcomes of pars repair surgery using pedicle screws and laminar hooks. *Methods*: This study was conducted on 22 patients with symptomatic lumbar spondylolysis. Curettage of the fibrocartilage in the defect and drilling of the sclerotic bone ends were done, followed by impaction of cancellous bone graft. Pedicle screws were inserted bilaterally in the corresponding pedicles and connected to a laminar hook via rods (screw-rod-hook fixation). The intensity of back pain and the functional outcome were assessed using the visual analog scale (VAS) and the Oswestry disability index (ODI). Plain radiographs were performed immediately postoperatively and after 3 and 6 months. CT scan was done at the final follow-up to assess pars healing. The mean follow-up period was 27 months. *Results*: The mean preoperative VAS and ODI were 7.4 ± 0.8 and 64.8 ± 6.7, which improved to 2.4 ± 0.8 and 20 ± 6 respectively at the final follow-up (*P* < 0.001). Healing of the defect was found in 19 patients at the final follow-up. Non-fusion with graft resorption was noticed in the remaining 3 cases (13.6%). However, postoperative VAS and ODI values improved even in the radiologically non-fused patients. *Level of evidence*: Therapeutic study, Level IV. *Conclusion*: Pars repair using pedicle screws and laminar hooks is a relatively simple yet effective procedure.

## Introduction

Lumbar spondylolysis is considered a common finding in adolescents and adults, with a reported prevalence of up to 6% [1]. Although usually asymptomatic and accidentally discovered, it could cause low back pain needing either conservative or surgical treatment [2]. Posterior spine fusion could be performed to treat symptomatic pars interarticularis defect after the failure of conservative treatment. However, it has disadvantages such as potential adjacent segment disease [3]. Pars repair via bone grafting, first introduced by Kimura [4] in 1968, aimed to restore the stability of the vertebra by promoting the union of the pars defect while preserving the motion of the spinal segment. Many techniques for pars repair since then have been introduced as the Scott wiring technique [5], Buck trans-isthmic screws [6], Morscher hook and screw construct [7], pedicle screws with segmental wiring technique [8], and rod-screw construct [9]. Tokuhashi and Matsuzaki proposed a repair technique where pedicle screws and laminar hooks were used (screw-rod-hook fixation) [10]. A significant difference can be found in the literature regarding the difficulty of surgical technique and postoperative results according to the method used [4–10]. Our study aims to elaborate on Tokuhashi’s study, primarily clarifying the clinical results and secondarily the radiological outcome, operative time, and blood loss in pars repair via his technique to decide on optimum surgery for symptomatic lumbar spondylolysis.

## Materials and methods

After Institutional Review Board approval, this study was conducted on 22 patients with symptomatic spondylolysis at either L4 or L5 (L4 in 5 cases and L5 in 17 cases). Informed consent was obtained. Fifteen were males and 7 females. The age of the patients ranged from 22 to 37 years with a mean of 29.4 ± 4.6 years. Indications for surgery included bilateral pars defect at L4 or L5, low back pain with no satisfactory response to conservative treatment for at least 6 months (medical treatment, physiotherapy, and lifestyle modification), positive response to local anesthetic infiltration in the pars defect and Pfirrmann’s [11] grade 1 or 2 intervertebral disc on magnetic resonance imaging (MRI) with no spondylolisthesis. Patients who underwent previous spine surgeries, those with neurological deficits or multi-level pars defects were excluded from the study. Medical history was obtained preoperatively from every patient as well as performing the detailed physical and neurological examination. Preoperative standing anteroposterior and lateral radiographs with flexion-extension views, in addition to computed tomography (CT) scan and MRI were done for all. The intensity of low back pain was assessed using the visual analog scale (VAS) [12] and the functional outcome was assessed using the Oswestry disability index (ODI) [13].

All patients were operated in prone position under general anesthesia using a radiolucent surgical table. A posterior midline vertical skin incision was used, with subperiosteal paraspinal muscles dissection. Curettage of the fibrocartilaginous tissue in the defect with exposure of the sclerotic bone ends was done. Drilling the sclerotic bone on either side of the defect was performed to promote healing with impaction of cancellous bone graft harvested from the posterior iliac crest into the defect. Titanium pedicle screws were inserted in the corresponding pedicles bilaterally and were connected on each side to a laminar hook via rods. Compression was applied between the screw and the corresponding hook before tightening the nuts. The position of the implants was checked intraoperatively by fluoroscopy, then the wound was closed routinely. Patients were encouraged to walk the next day after surgery without a brace.

Operative time and blood loss were documented along with any intraoperative or postoperative complications. Radiographs were performed immediately postoperatively and after 3 and 6 months to check implant position. The mean follow-up period was 27 months. CT scan was done at the final follow-up to assess the healing of the pars. Both VAS for back pain and ODI were recorded at the final follow-up and compared with their respective preoperative values.

Statistical analysis was done using the SPSS program version 17.0 (IBM SPSS Statistics, Chicago, Illinois). Data were assessed by paired *t*-test where *P* values less than 0.05 were considered statistically significant and those less than 0.001 were statistically highly significant.

## Results

**Figure 1:**
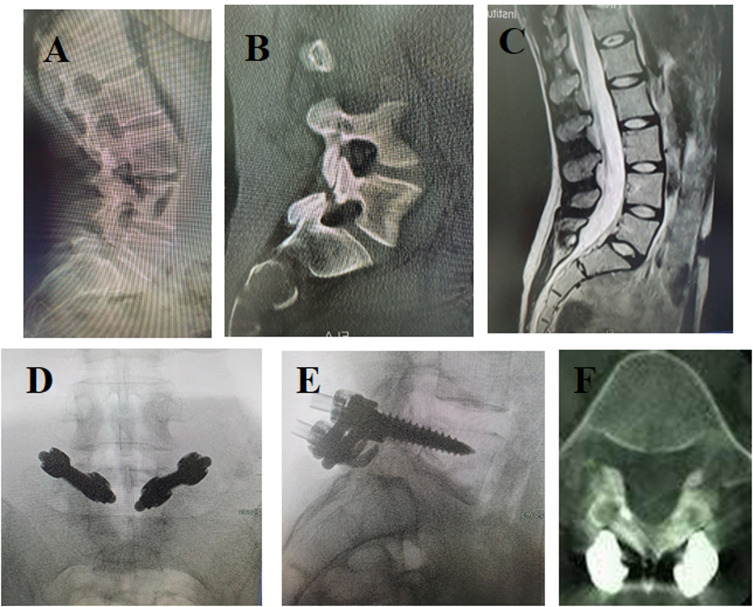
Twenty-six years old female with symptomatic spondylolysis performed pars repair after failed conservative treatment for 6 months. (A) Preoperative plain X-ray, (B) preoperative CT, and (C) preoperative MRI showing spondylolysis at L5. Pedicle screw hook fixation was performed. (D, E) Intraoperative anteroposterior and lateral images. (F) CT at final follow-up showing healing of the defect.

The operative time ranged between 135 and 185 min with a mean operative time of 158.6 ± 15.8 min. The blood loss ranged between 315 and 410 cc with a mean blood loss of 361.8 ± 25.2 cc. The mean preoperative VAS for low back pain was 7.4 ± 0.8 which improved to 2.4 ± 0.8, at the final follow-up. VAS improvement was statistically highly significant with a *P-*value < 0.001. The mean preoperative ODI was 64.8 ± 6.7. The mean ODI improved to 20 ± 6 at the final follow-up. The improvement in values was statistically highly significant, with *P*-value < 0.001. Healing of the defect was found among 19 patients at the final follow-up. No union was noticed in the other 3 cases (13.6%) with resorption of the bone graft. However, postoperative VAS and ODI values improved even in the radiologically non-united patients. There were no cases with postoperative infection or neurological deficit. No metal failure was observed during the follow-up period (Figure 1). Two patients suffered from transient lower limb pain (on the side of bone graft harvest), which disappeared during the first postoperative week (Table 1).

**Table 1:** Demographic data, operative time, blood loss, fusion status, VAS scoring, ODI scoring

Patient	Age	Sex	Operative time	Blood loss (mL)	Fusion status	Preoperative VAS	Last follow-up VAS	Preoperative ODI	Last follow-up ODI
1	29	Male	150	330	Fused	7	2	58	18
2	31	Male	140	340	Fused	8	4	70	32
3	22	Male	175	365	Fused	6	1	56	12
4	30	Male	140	360	Not fused	8	2	64	20
5	27	Female	155	335	Fused	7	1	60	10
6	36	Female	135	345	Not fused	9	3	76	24
7	33	Male	160	370	Fused	8	3	64	22
8	23	Male	185	390	Fused	6	2	56	16
9	34	Female	135	315	Fused	8	2	70	18
10	22	Male	165	340	Fused	7	4	62	30
11	29	Female	140	360	Not fused	8	4	68	28
12	34	Male	140	325	Fused	8	3	62	24
13	37	Female	180	400	Fused	8	2	66	20
14	35	Male	160	370	Fused	6	2	54	14
15	26	Female	170	400	Fused	8	2	72	22
16	23	Male	145	350	Fused	7	3	70	26
17	34	Male	170	390	Fused	7	2	66	16
18	26	Female	180	370	Fused	7	1	56	8
19	25	Male	170	370	Fused	9	2	78	18
20	33	Male	155	375	Fused	8	2	74	16
21	28	Male	160	350	Fused	7	3	60	22
22	30	Male	180	410	Fused	7	3	64	24

## Discussion

Pars interarticularis defect referred to as spondylolysis is not a rare pathology with a prevalence of up to 6% [1]. L5 followed by L4 vertebrae are usually the most commonly affected levels [14]. Spinal fusion was frequently used as a surgical treatment for spondylolysis even if not accompanied by spondylolisthesis or degenerative disc disease [15, 16]. However, the technique has well-known drawbacks such as loss of mobile spinal segment and affection of adjacent level [17, 18]. As a result, the idea of pars repair was taken more into consideration [4–10].

In the present study, authors used the screw rod hook fixation technique proposed by Tokuhashi and Matsuzaki [10]. It was proved in the study by Deguchi et al. [19] to be rigid with minimal micro-motion across the pars defect. The technique is relatively easy and allows adequate compression on the defect site [10]. In our study, the technique improved of low back pain and functional recovery with high healing rates of the defect and no implant failure. Clinical improvement was noted even in the cases which showed radiological non-union at final follow-up.

As a limitation to the present study, the surgery was performed on patients with narrow sets of criteria. Only patients with Pfirrmann’s [11] grade 1 or 2 intervertebral disc at MRI with no spondylolisthesis were included. In future studies, extending the indications of the technique to include Pfirrmann’s [11] grade 3 intervertebral disc in MRI with a mild form of spondylolisthesis may allow us to have a study on a larger number of patients correlating between the clinical satisfaction of the patients and the pre-operative X-ray and MRI findings of the corresponding intervertebral disc.

Tokuhashi and Matsuzaki [10] performed the surgery on 6 patients. All patients experienced significant pain relief, and healing of the defect occurred in 100% of patients with no instrumentation failure. Another study performed on 16 patients by Kakiuchi [20] showed similar results with 100% defect healing as seen on oblique radiographs, and 81% of patients were pain-free at the end of follow-up. A study performed by Roca et al. [21] on 19 patients revealed that clinical outcome was excellent to good in 79% of patients. However, the percentage of successful defect healing was 63% on CT scans, as no healing was noted in patients above 20 years. When limited to patients below 20 years of age, the rate of healing improved to 92% [21]. Comparable results were obtained in our study with a larger number of patients (22 patients) where healing of the defect occurred in 86.4% of patients and clinical improvement was achieved in all patients at final follow-up. The clinical improvement and rates of bone healing in different studies are summarized in (Tables 2 and 3) [10, 20–25]. VAS score improved significantly in all except the study of Ishida et al. [25] due to a small number of patients.

**Table 2:** Preoperative VAS for low back pain and ODI score before surgery and at final follow-up in different studies performing pars repair by the technique.

	Patients (*n*)	VAS Preop	VAS final follow-up	*P*-value	ODI Preop	ODI final follow-up	*P*-value
Debusscher [22]	<30 years: 12	7.75 ± 1	1.7 ± 0.9	<0.05	71 ± 7	11.3 ± 5.5	<0.05
Debusscher [22]	>30 years: 11	7.8 ± 0.75	2.7 ± 1.2	<0.05	74.2 ± 6.2	19.8 ± 10.3	<0.05
Shin [23]	23	4.9 ± 1.4	2.2 ± 0.9	<0.05	50.6 ± 16.6	24.6 ± 13.4	<0.05
Raudenbush [24]	9	5.6	1.2	–	–	–	–
Ishida [25]	2	8 ± 0	4 ± 0	–	–	–	–
Present study	22	7.4 ± 0.8	2.4 ± 0.8	<0.001	64.8 ± 6.7	20 ± 6	<0.001

**Table 3:** Bone union at the pars defect in different studies performing the technique.

	Total patients (*n*)	Follow-up period (months)	Bone union
Tokuhashi [10]	6	12	100%
Kakiuchi [20]	16	25	100%
Debusscher [22]	23	12	91%
Shin [23]	23	37	78.3%
Raudenbush [24]	9	11.9	77.8%
Ishida [25]	5	12	40%
Roca [21]	<20 years: 13	12	92%
	>20 years: 6	12	0%
Present study	22	27	96.4%

Disc degeneration or the presence of spondylolisthesis are assumed to be contraindications to pars repair [26, 27]. A study by Louis [28] tried to extend the indications of pars repair to include patients with up to one cm slip and loss of up to 1/3 of the disc height. He reported that the results were excellent or good as regards pain in 86% of patients, and 88% of patients were able to return to heavy work [28]. The present study included only patients with Pfirrmann’s [11] grade 1 or 2 intervertebral disc at MRI with no spondylolisthesis. Some studies also reported that failure of healing of the defect is usually associated with poor results [21]. This correlation was not recognized in the present study as the patients who did not show healing on final follow-up CT also showed clinical improvement. However, it must be taken into consideration the low number (3 patients only) of non-united patients.

Older techniques of pars repair have their drawbacks such as the Kimura technique, where no instruments were used depending on external cast immobilization [4]. The later technique of Scott [5] wiring had technical difficulties that could lead to nerve root injury or excessive bleeding with the possibility of wire breakage. Inserting the screw through the pars interarticularis described by Buck [6] remains technically demanding [29]. Ishida reported on a case of dural tear during sublaminar hook insertion when performing pedicle screw hook fixation [25]. The complication was minimal in this study. No implant failure was recorded at the final follow-up. Two patients suffered from transient lower limb pain (on the side of bone graft harvest), which disappeared within 1 week.

## Conclusion

Pars interarticularis repair using pedicle screws and laminar hooks fixation technique is an effective and relatively simple.

## Conflict of interest

The authors declare that they have no relevant financial or non-financial interests to report.

## Funding

This research did not receive any specific funding.

## Ethical approval

The study was conducted after approval of the ethics committee of our institute (IRB No. FMASU 1967/2021)

## Informed consent

Written informed consent was obtained from all patients and/or families

## Author’s contributions

All authors contributed to the study’s conception and design, material preparation, data collection, and analysis. The first draft of the manuscript was written by first author and all authors commented on previous versions of the manuscript. All authors read and approved the final manuscript.

## Data availability

The datasets generated and analyzed during the current study are available from the corresponding author on reasonable request.
